# A Mediterranean diet is associated with improved total antioxidant content of human breast milk and infant urine

**DOI:** 10.1186/s12937-023-00841-0

**Published:** 2023-02-23

**Authors:** Samira Karbasi, Malihe Mohamadian, Mohsen Naseri, Zahra Khorasanchi, Asghar Zarban, Afsane Bahrami, Gordon A. Ferns

**Affiliations:** 1 Department of Molecular Medicine, School of Medicine, Cardiovascular Diseases Research Center, Birjand University of Medical Sciences, Birjand, Iran; 2 Cellular and Molecular Research Center, Department of Molecular Medicine, Birjand University of Medical Sciences, Birjand, Iran; 3 Department of Nutrition, School of Medicine, Mashhad University of Medical Sciences, Mashhad, Iran; 4 Cardiovascular Diseases Research Center, Birjand University of Medical Sciences, Birjand, Iran; 5 Clinical Biochemistry Department, Faculty of Medicine, Birjand University of Medical Sciences, Birjand, Iran; 6 Clinical Research Development Unit, Imam Reza Hospital, Faculty of Medicine, Mashhad University of Medical Sciences, Mashhad, Iran; 7 Clinical Research Development Unit of Akbar Hospital, Mashhad University of Medical Sciences, Mashhad, Iran; 8 Division of Medical Education, Brighton & Sussex Medical School, Falmer, Brighton, BN1 9PH Sussex UK

**Keywords:** Mediterranean diet, Antioxidant content, Mothers, Infant urine, Breast milk macronutrients

## Abstract

**Background:**

The composition of breast milk (BM) is dynamic and can be influenced by maternal variables that include the diet and nutritional status. This study examined the association between maternal adherence to a Mediterranean diet (MedDiet) and total antioxidant content of BM and infant urine.

**Methods:**

We collected 350 BM samples from mothers and urine samples from their infants. The dietary intakes of the mothers were recorded using a validated 65 items-food frequency questionnaire (FFQ). The total antioxidant status of the samples was assessed using the ferric reducing/antioxidant power (FRAP), the 1, 1-diphenyl-2-picrylhydrazyl (DPPH), thiobarbituric acid reactive substances (TBARS), and thiol quantification assays. Milk protein, calcium, and triglyceride (TG) were also determined using standard biochemical kits.

**Results:**

Subjects with the highest MedDiet scores were among the women in the highest tertile (T3) for consumption of dietary fiber, fruits, vegetables, nuts, legumes, and seeds, low-fat dairy, whole grains, and the lowest consumption of red meat, sweetened beverages, and sodium, compared to women in the first tertile (T1) with the lowest MedDiet scores. After adjustment for potential confounders, the individuals in the highest tertile for MedDiet score had a significantly higher level of milk DPPH, and infant urinary DPPH than the lowest tertile and had a significantly higher level of milk protein, FRAP and infant urinary FRAP compared to the T2 (*P* < 0.05). In addition, the mothers in the T3 for the MedDiet pattern had a significantly lower level of milk TG compared to those within the T1 (*P* < 0.05).

**Conclusion:**

Our findings show that a high maternal adherence to a MedDiet can affect BM composition and their infants' urine.

## Introduction

Exclusive breastfeeding has been considered the most optimum nutritional source for the first six months of life. This has been shown in numerous reported short- and long-term studies on breast milk (BM) on the biological functions and development of the infant [1–3]. According to these studies, breast feeding has profound effects on the child's and mothers' cognition, behaviors, and mental health in addition to providing a meal at the breast [4]. Evidence suggests that BM has prophylactic effects against sudden infant death syndrome (SIDS) [5], cardiovascular disease (CVD) [6], respiratory infections [7], allergic pathologies [8], gastrointestinal disorders [9, 10], obesity, and diabetes [11]. Indeed, these protective effects of BM are associated with a wide range of bioactive factors, including vitamins, hormones, fatty acids (FAs), essential amino acids, minerals, antioxidants, growth factors, and immunoglobulins, which play regulatory and immunomodulatory roles [12].

Immediately after birth, neonates are at risk of an increased production of reactive oxygen species (ROS) due to a rapid transition from the intrauterine to an environment with higher concentrations of oxygen [13]. Oxidative stress, resulting from an imbalance between the ROS level and the potential of cellular antioxidant protection systems, is an underlying mechanism in the pathogenesis of many neonatal diseases such as necrotizing enterocolitis, bronchopulmonary dysplasia, intraventricular hemorrhage, renal failure, and premature retinopathy [14, 15]. Therefore, antioxidants are considered to be an important component of a newborns’ diet. BM contains enzymatic antioxidant enzymes such as glutathione peroxidase (GPx), catalase, and superoxide dismutase (SOD) as well as non-enzymatic antioxidants such as vitamins C, E, and A, carotenoids, lactoferrin, α-tocopherol, and ascorbate which protects neonates during lactation [3, 16, 17].

The American Dietetic Association has recommended that women who are ready to have children should maintain a healthy nutrient intake by leading a lifestyle that promotes women's health and lowers the risk of birth defects, inadequate neonatal growth and development, and chronic health issues in their offspring [18]. The main elements of lifestyle that promotes health in the mother, include: appropriate weight gain, optimal physical activity, intake of a range of healthy foods, and adequate and timely mineral and vitamin supplements [19]. BM content is not consistent and varies depending on the mother's dietary, season, and stage of breastfeeding [20]. In particular, the total antioxidant capacity (TAC) of BM as well as its concentrations of FAs and vitamins A, C, B-6, and B-12 have been shown to reflect the intake of these nutrients through the maternal diet [21, 22]. In order to have a beneficial impact on the health of both the mother and the infant, it is essential to adopt appropriate dietary habits throughout gestation lactation [23].

A Mediterranean diet (MedDiet) is a well-established dietary pattern, that was characterised in the early 1960s [24] as comprising a high consumption of plant-based foods such as fruit, vegetables, legumes, grains, nuts, and especially olive oil, together with moderate consumption of poultry, eggs, seafood, and dairy products (especially cheese and yogurt) and low consumption of red and processed meats [25, 26]. Since a MedDiet provides a significant source of vitamins, probiotics, minerals, and polyphenols, it is proposed to promote physical and mental health and alleviates the risk of CVD, metabolic disorders, certain types of malignancies, and more [27]. Furthermore, the correlation found between lower score and relatively short gestation endorses new findings on the advantages of the MedDiet in the prevention of early deliveries, as well as the importance of a sufficient supply of folates and Fe, among many other micronutrients, for the proper development of the fetus [28, 29]. As a result, a healthy diet is essential across the reproductive years and after pregnancy to ensure the health of both mother and child [30].

The previous research on the relationship between the diets of breastfeeding women and the oxidant-antioxidant content of human milk has a number of limitations, including the small sample size of studies. Also, given the prominence of BM composition in the development of infants, the association between maternal adherence to the MedDiet and BM composition was investigated in this study.

## Material and methods

### Study design and participants

This cross-sectional study was carried out between January and February 2021, in southern Khorasan, Birjand, Iran. This study was approved by the Medical Ethics Committee of Birjand University of Medical Sciences, Birjand, Iran. Following a stratified cluster sampling strategy, 350 breastfeeding mothers aged 20 to 35 years were randomly selected from four health clinics in 4 different areas of the city. All women included had a newborn aged 1–6 months, and women who had chronic conditions (e.g., hepatic or renal failure, CVD, autoimmune disease, malignancy, and anorexia nervosa), a history of taking anti-inflammatory, anti-diabetic, anti-depressant, or anti-obesity drugs in the last 6 months, were overweight or too thin, used tobacco, or took supplements were not included in the study. Before any data collection, written informed consent was signed by each participant. Each mother was requested to provide two samples of BM in 20-ml volumes, manually expressed from the primary breastfeeding, at the beginning of the day (7 a.m. and 10 a.m.) before the infant was fed. Additionally, a urine sample of 10 ml was collected from each infant using a urine bag. In health centers, samples were collected into sterile tubes and sent to the laboratory on dry ice. The samples were freeze-dried and kept at -80°C until tested.

### Covariate’s measurement

Health care professionals and a nurse interviewer collected information about participants' demographic characteristics such as name, age, gender, economic status, marital status, education level, height (cm), weight (kg), body mass index (BMI) (kg/m 2), blood pressure (BP), infant age, sex, weight, head circumference (cm) and medical history. BMI was determined by dividing weight (kilograms) by height (meters) squared. The height and circumference of the infant's head were recorded to the nearest millimeter with a measuring tape. Digital scales were used to measure weight to the nearest 0.1 kg. BP was measured in the left arm of individuals who were sitting and at rest after 15 min using a mercury sphygmomanometer. This was repeated twice in the same manner. If the results varied by more than 15 mmHg in diastolic blood pressure (DBP) or more than 25 mmHg in systolic blood pressure (SBP), we performed a third test and averaged the two closest readings (25).

### Determination of total antioxidant status

#### Total antioxidant capacity measured by Ferric reducing antioxidant power (FRAP) assay

To measure the TAC of BM and infant urine, the FRAP assay was conducted as described by Benzie and Strain with slight modification [31]. First, FRAP reagent was made by combining 300 mmol/L acetate buffer, 10 mmol/L 2,4,6-tripyridyl-s-triazine (TPTZ), and 20 mmol/L FeCl3 with volume ratios of 10:1:1 and at pH 3.6. After mixing 250 µL of the FRAP reagent with a 10 µL BM sample, standard (FeSO_4_) or blank the reaction mixture was further incubated at 37 °C for 10 min. Reduction of the ferric-TPTZ complex to the ferrous-TPTZ by an antioxidant agent renders an intense blue color, which is measurable through the absorbance value at 593 nm [32]. Five different concentrations of FeSO_4_.7H_2_O were used for calibration and standard curve preparation. To ensure accuracy, all tests were performed twice and the average was calculated. This method validated for BM previously [33, 34].

#### Radical scavenging activity evaluated by 2, 2′-diphenyl-1-picrylhydrazyl (DPPH) assay

The potential of BM and infant urine samples to scavenging of DPPH radicals was determined by a modified form of the method proposed by Brand-Williams et al. [35]. Briefly, 50 μl of each BM sample was added to 950 μl of DPPH in methanol solution (100 mM) and then incubated at room temperature for 15 min. The resultant content was centrifuged at 4000 g for 5 min, and its absorbance was determined at 517 nm using a spectrophotometer. An methanolic solution of DPPH (100 mM) was applied as a control. The scavenging activity of DPPH radicals was calculated as follows: Scavenging activity (%) = [(absorbance of the control – absorbance of the sample)/absorbance of the control] * 100. Each test was performed in duplicate and the results are shown in mmol eq. Trolox/L. This method validated for breast milk previously [33, 34].

#### Thiobarbituric acid reactive substances (TBARS) assay

TBARS assay is a common method for measuring lipid peroxidation products. The reaction of malondialdehyde (MDA), the main marker of lipid peroxidation, with thiobarbituric acid (TBA) leads to the formation of a pink complex (TBARS) that can be detected spectrophotometrically [36]. One ml of TBA / HCl reagent was added to 100 μl of each BM and infant urine sample, and the resultant mixture was incubated in a hot water bath for 25 min and then cooled. To improve the sensitivity of the assay, the TBARS adducts were precipitated with 1 ml of N-butanol adducts and the solution was centrifuged for 10 min at 1500 g at 4° C. 1,1,3,3, tetra-methoxy propane was used as the standard. Later, the fluorescence intensity of the samples was read at excitation/emission wavelengths of 515/553 nm.

#### Thiol quantification assay

For detecting free sulfhydryl groups in BM samples, the chromogenic Ellman’s reagent, or 5,5’-dithiol-bis-(2-nitrobenzoic acid) (DTNB) was used. Fifty microliters of BM samples were mixed with 1ml of Tris/EDTA buffer, 50 µl of 10 mM DTNB solution, and 650 µL N-butanol and then centrifuged for 3 min at 3000 g. Following the reduction of the highly oxidizing disulfide bond in DTNB by free thiols, 5-thio-2-nitrobenzoic acid (TNB), a yellow product with strong absorbance at 412 nm. is released [37]. The mixture was incubated at room temperature for 15 minutes and the net adsorption was achieved by subtracting the absorbance of a DTNB blank (containing methanol instead of the sample and reduced glutathione as the T-SH group standard) from the apparent absorbance.

#### Milk calcium, protein, and triglyceride

The results of each photometric analysis were assessed at 37 °C using a plate reader (EpochTM, BioTek, Winooski, VT, USA). All absorbance data were evaluated using monochromatic readings [38]. Calcium was calculated using the Arsenazo III kit (Pars Azmoon, Tehran, Iran) in accordance with the user's instructions. At neutral pH, calcium forms a compound with Arsenazo III, and the color intensity is proportionate to the calcium amount in the sample [39]. All samples' and blanks' absorbance levels were measured at 660 nm. Ten µl of human milk sample and 1 ml of color reagent were used in triplicate for the Bradford protein test. The components were combined for 30 s, and the absorbance values of each sample and the blank (10 µl of each human milk sample and 1 ml of distilled water duplicate) were assessed using a microplate reader at 595 nm after 5 min of incubation at room temperature [40]. A Pars Azmoon® kit (Tehran, Iran) was used to measure the milk's triglyceride levels. It is an assay that makes use of enzymatic hydrolysis, quantification by evaluating absorbance at 546 nm, and data analysis in mg/dL. 1 mL of the triglyceride reagent was added to an aliquot of 10 µl of the BM sample (diluted 1:10), vortexed, and then incubated for 30 min at 37° C. All samples and blanks (1 ml of distilled water and 10 µl samples) had their absorbance measured at 546 nm.

### Assessment of dietary intake

Participants habitual diet for the previous year was recorded following face-to-face interviews using a validated semi-quantitative food frequency questionnaire (FFQ) consisting of 65 food items [41]. This FFQ has been previously validated in the Iranian population [42]. Individual nutritional intakes were evaluated using Dietplan6 software (Forest field Software Ltd., Horsham, UK) [43].

The degree of maternal adherence to the MedDiet was determined using the scale developed by Trichopoulou et al. [44]. Accordingly, a value of 0 or 1 was assigned to each component of the diet, and the median was used as a cut-off. For fruits, vegetables, nuts, grain, legumes, fish, and seafood as well as the ratio of monounsaturated to saturated fatty acids (MUFA/SFA), lower than median intakes were assigned a value of 0, while intakes equal to or greater than the median were assigned a value of 1. For meat and dairy products, the scoring trend was inverted. Due to the illegality of alcohol consumption in Iran, it cannot be evaluated as a food item in the current study. The total MedDiet score is calculated by adding the score of each group and is between 0 (minimal adherence) and 8 (Maximal adherence).

### Statistical analysis

Data analysis was performed using the statistical package for social sciences (SPSS Inc. Chicago, IL, version 16.0). The acquired MedDiet scores were categorized into tertiles (low, medium, and high adherence). Kolmogorov-Smirnov analysis was employed to assess the variables’ normality. To study continuous variables with normal distribution among tertiles, a one-way ANOVA test was used. Pearson correlation test was conducted to evaluate the relationship between MedDiet score and components of BM. Multivariate logistic regression analysis was also undertaken to specify the association between the MedDiet adherence and components of BM. Logistic regression models were adjusted for age, BMI, and energy consumption. A *P*-value <0.05 was determined to be statistically significant.

## Results

The 350 women had an average age of 29.5±5.9 years. They were divided into three groups based on the tertiles of their MedDiet scores: T1 (lower adherence; *n*= 118), T2 (medium adherence; *n*= 112), and T3 (higher adherence; *n*= 120). There was no significant relationship between the general characteristics and anthropometric parameters of the participants (Table 1), including maternal age, BMI, SBP, and baby age and weight and head circumference, but mother DBP was shown to be significantly higher in the lowest tertile (T1) than in the highest tertile (T3) of MedDiet adherence (*P*> 0.05).

**Table 1:** Demographic, anthropometric and clinical characteristics of the participants across the three tertiles of adherence to the MedDiet (T1, T2 and T3)

Variables	**T1** 118(33.71%)	**T2** 112(32%)	**T3** 120(34.28%)	*P* value^a^
Maternal				
Age (y)	29.9 ± 5	29.4 ± 6.1	28.5 ± 5.3	0.25
SBP (mmHg)	101 ± 0.7	101 ± 1.2	103 ± 1.0	0.19
DBP (mmHg)	52 ± 3.4	32 ± 3.6	19 ± 2.0	**0.001**
BMI (Kg/m2)	24.4 ± 4	23.5 ± 4.2	24.3 ± 3.0	0.38
Infant				
Age (days)	139.5 ± 55.7	147.2 ± 50.0	148.7 ± 33.8	0.39
Sex (male), n (%)	128 (55.7%)	134 (53.6%)	80 (46.5%)	0.13
Weight (Kg)	7.1 ± 1.6	6.9 ± 1.4	7.3 ± 2.6	0.24
Head circumference (cm)	38.7 ± 23	39.9 ± 34	39.7 ± 28	0.13

Data presented as Mean ± SD

*BMI* Body mass index, *SBP* Systolic blood pressure, *DBP* Diastolic blood pressure, *DP* Dietary pattern

^a^*p*-value from analysis of the variance (ANOVA) for groups comparison

Food components for mothers in the three tertiles in terms of MedDiet score (Table 2), indicate a significant association between certain dietary factors and MedDiet adherence rate (*P* > 0.05). The amount of MUFA, SFA, fruits, legumes, nuts, fish and seafood, and MedDiet adherence were significantly higher in T3 compared to T1. The consumption of dairy and meat products was significantly lower in subjects in T3 compared to T1 (*P* < 0.05).

**Table 2:** Comparison of participant dietary intakes across the three tertiles of adherence to the MedDiet scores

***Food groups (g/1000 kcal)***	MedDiet Adherence			*P*-value
	**T1** 118(33.71%)	**T2** 112(32%)	**T3** 120(34.28%)	
Vegetables	129.1 ± 147.1	176.1 ± 116.0	194.6 ± 126.8	**0.001**
Fruits	68.0 ± 64.3	110.5 ± 32.4	125.5 ± 78.6	** < 0.001**
Legumes and nuts	8.1 ± 10.1	9.5 ± 8.7	11.5 ± 9.4	**0.003**
Grains	133.9 ± 96.5	187.3 ± 68.8	233.6 ± 84.3	** < 0.001**
Fish and seafood	4.9 ± 10.7	10.5 ± 3.1	11.4 ± 7.1	**0.001**
MUFA/SFA	0.26 ± 0.44	0.56 ± 0.21	0.90 ± 0.33	** < 0.001**
Dairy products	198 ± 176	102 ± 115	117 ± 175	**0.006**
Meat products	58.4 ± 33.2	24.3 ± 11.7	34.5 ± 16.1	**0.011**

Data presented as Mean ± SD and adjusted for energy intake

T1 represents low compliance and T3 a high compliance with a MedDiet

*MUFA* Monounsaturated fatty acid, *SFA* Saturated fatty acid, *PUFA* Polyunsaturated fatty acid

*p*-value obtained from independent sample-test

The amount of BM anti-oxidants and infant urinary anti-oxidants in relation to the pattern of the MedDiet is shown in Table 3. The individuals in the T3 for the MedDiet pattern had a significantly higher level of milk DPPH, and infant urinary DPPH than the T1 and had a significantly higher level of milk protein, FRAP and infant urinary FRAP compared to the T2 (*P* < 0.05). In addition, the mothers with the T3 to the MedDiet pattern had a significantly lower level of milk TG compared to those with the T1(*P* < 0.05).

**Table 3:** BM composition and infant urinary anti-oxidant status across the three tertiles of adherence to the MedDiet score

Variables	**T1** 118(33.71%)	**T2** 112(32%)	**T3** 120(34.28%)	*P* value^a^
Maternal milk				
DPPH (µmol eq. Trolox/L)	306 ± 66	348 ± 95	336 ± 54	**0.001**^**γ**^
FRAP (µmol /L)	540 ± 150	553 ± 150	558 ± 164	**0.046**^**β**^
MDA (μmol TBARs/L)	0.127 ± 0.08	0.115 ± 0.07	0.128 ± 0.08	0.35
Thiol (µmol/L)	79 ± 23	79 ± 19	75 ± 16	0.67
Calcium (mg/dL)	8.79 ± 1.18	8.81 ± 1.20	9.08 ± 1.10	0.11
Protein (g/dL)	1.38 ± 0.66	1.47 ± 0.68	1.39 ± 0.73	**0.009**^**β**^
Triglyceride (mg/dL)	4.45 ± 1.80	4.21 ± 1.27	4.02 ± 1.58	**0.048**^**γ**^
Infant urine				
FRAP (µmol /L)	22.2 ± 18	20.5 ± 15.5	27.1 ± 13.6	**0.02**^**β**^
DPPH (µmol eq. Trolox/L)	10 ± 8.1	9.3 ± 7.4	13.7 ± 6.4	**0.014**^**γ**^
MDA (μmol TBARs/L)	1.75 ± 1.5	1.72 ± 1.8	2.1 ± 1.1	0.42

^**α**^Significant between groups 1 and 2

^**β**^Significant between groups 2 and

^**γ**^Significant between groups 1 and 3

*p*-value from analysis of the variance (ANOVA) and post hoc Tukey for groups comparison

The odds ratios for BM contents and infant urinary contents across tertiles of MedDiet are shown using adjusted models in Table 4. The first model, BM contents regression was adjusted for mother age, and energy intake. Further BM contents adjustments were made for mother SBP, DBP and BMI. The first model, infant urine contents regression was adjusted for infant age, and sex. Further infant urine contents adjustments were made for infant weight and head circumference. Using the tow-adjusted model, after controlling for additional potential confounders, adhering to the MedDiet was associated with increased odds of milk DPPH (OR = 1.43, 95% CI: 1.23–1.56; *P*-value < 0.05), FRAP (OR = 1.19, 95% CI: 1.12–1.28; *P*-value < 0.05), protein (OR = 2.10, 95% CI: 1.04–3.17; *P*-value < 0.01), and infant urinary DPPH (OR = 1.70, 95% CI: 1.48–2.11; *P*-value < 0.05) and FRAP (OR = 1.11, 95% CI: 1.07–1.18; *P*-value < 0.05) and decreased odds of milk TG (OR = 0.62, 95% CI: 0.52–0.85; *P*-value < 0.05).

**Table 4:** Multivariate adjusted odds ratios (95% CIs) for BM contents and infant urinary contents across the three tertiles of adherence to the MedDiet scores

**Variables**	**Crude**	**Model** I	**Model** II
Maternal milk^a^			
DPPH (µmol eq. Trolox/L)			
T1	1.0	1.0	1.0
T2	1.23(1.11–1.42) ^**^	1.03(1.01–1.11) ^**^	0.99(0.9–0.99)
T3	1.20(1.03–1.42) ^*^	1.29(1.12–1.49) ^*^	1.43(1.23–1.56) ^*^
FRAP (µmol /L)			
T1	1.0	1.0	1.0
T2	1.10(0.97–1.23)	1.11(0.97–1.20)	1.03(0.95–1.03)
T3	1.20(1.11–1.35) ^*^	1.17(1.08–1.25) ^*^	1.19(1.12–1.28) ^*^
Protein (g/dL)			
T1	1.0	1.0	1.0
T2	0.88(0.82–0.96)	0.83(0.78–0.88)	0.90(0.89–0.97)
T3	2.19(1.25–3.36) ^**^	2.14(1.19–3.25) ^**^	2.10(1.04–3.17) ^**^
Triglyceride (mg/dL)			
T1	1.0	1.0	1.0
T2	0.22(0.11–0.51)	0.26(0.14–0.54)	0.32(0.20–0.57)
T3	0.53(0.34–0.77) ^*^	0.59(0.42–0.82) ^*^	0.62(0.52–0.85) ^*^
Infant urine^b^			
FRAP (µmol /L)			
T1	1.0	1.0	1.0
T2	0.97(0.97–1.02)	0.88(0.87–0.99)	0.96(0.93–1.03)
T3	1.13(1.11–1.23) ^*^	1.15(1.14–1.25) ^*^	1.11(1.07–1.18) ^*^
DPPH (µmol eq. Trolox/L)			
T1	1.0	1.0	1.0
T2	0.95(0.96–1.02)	0.82(0.76–0.90)	0.75(0.63–0.88)
T3	1.46(1.27–1.68) ^*^	1.68(1.35–1.89) ^*^	1.70(1.48–2.11) ^*^

T1 was considered as reference group

^a^Breast milk was adjusted in Model I: for mother age, and energy intake and in Model II additionally, adjusted for mother SBP, DBP and BMI

^b^Infant urine was adjusted in Model I for infant age, and sex and in Model II additionally, for infant weight and head circumference

^*^p < 0.05

^**^p < 0.01

^***^p < 0.001

## Discussion

We have investigated the effect of maternal adherence to the MedDiet on their milk composition and the resulting influence on infant urine. According to our findings there was a significantly higher level of DPPH and FRAP in the BM of mothers who had a high score for MedDiet intake. We also found a positive and significant correlation between MedDiet scores and infant urinary DPPH and FRAP levels. A potential indicator for assessing the quality of a diet is an integrated measurement of all antioxidants present in plasma and body fluids, known as the TAC [45]. Indeed, the consumption of vegetables, fruits, grains, and olive oil in the MedDiet ensures a high intake of vitamins B-6, B-12, C and E, β-carotene, polyphenols, folic acid, and minerals, all of which have obvious antioxidant effects. Even compared to supplements, the MedDiet is known to be a better approach to delivering antioxidants due to the synergism of various antioxidants [46]. Many studies have considered the MedDiet as a high TAC diet, which is largely due to its antioxidant properties as well as its positive effect on lipid metabolism [47–49]. A MedDiet intervention in healthy subjects demonstrated an 11% increase in serum TAC levels as well as a 19% decrease in oxidized LDL cholesterol levels in the highest tertile of the MedDiet score compared to the lowest one [46]. The PREDIMED study reported a significant increase in plasma FRAP and total radical-trapping antioxidant parameter (TRAP) following a one-year intervention with MedDiet + virgin olive oil or nuts in subjects at high risk for CVD [50]. Previously, Zarban et al. also revealed a far higher TAC in colostrum compared to transitional and mature milk using FRAP and DPPH assays. Moreover, a significant relationship was reported between the results of these two tests as well as the antioxidant capacity of BM and maternal plasma [33]. Previous evidence has also shown that the TAC of transitional and mature milk as well as urine infant may depend on the maternal dietary intakes of vitamins A, E, and C, β-carotene, and vegetables during the third trimester of pregnancy [34, 51–54]. A recent comparative study found that the BM of mothers adhering to the MedDiet contained twice as many phenolic compounds as infant formula. However, MedDiet intervention had no effect on total phenolic compound content (TPC) and TAC of the BM during the lactation stages [55].

According to our findings there was a significantly lower TG levels, as well as an increase in protein content in the BM of women who scored higher on the MedDiet assessment. On the other hand, the macronutrient contents of BM, namely proteins, carbohydrates, and lipids, seem to be less sensitive to maternal diet [56]. However, the association between the BM’s FA profile, as its main source of energy, and maternal diet has been evaluated in several studies. One study found an increment in total FAs levels, specially SFAs and MUFAs, as well as a decrease in TAC in BM of mothers who had a vegetable and fruit-rich diet than the MedDiet [17]. Also, in the BM secreted by the Croatian women under the MedDiet, the MUFAs, SFAs, and unsaturated fatty acids (UFAs) accounted for 42.26%, 34.95%, and 20.01% of total FAs, respectively. However, low intake of n − 3 long-chain polyunsaturated FAs (especially docosahexaenoic acid) of the MedDiet and consequently their low concentrations in BM is an issue of concern [57]. Our results are consistent with the evidence from other studies on the effect of MedDiet on TGs. Following the MedDiet intervention, alone or supplemented by walnuts, a decrease in the plasma content of TGs and TG-rich lipoprotein (TRL) was observed compared to a low-fat control diet [58–60]. The protein content of BM is vital for the physical growth and brain development of infants. Previous studies have shown that the BM protein levels vary depending on the lactation course but are less affected by the maternal diet [61]. However, Debski et al. have reported that the BM protein concentration in vegetarian and non-vegetarian mothers was 10.2 ± 1.4 g/100 ml and 9.9 ± 1.1 g/100 ml, respectively [62]. On the contrary, Huang and colleagues reported a negative correlation between protein concentrations in BM and more adherence to a pattern of high intake of vegetables, legumes, and low intake of poultry, red meat, and eggs [63]. However, it is thought that the increase in BM’s protein levels following more maternal adherence to MedDiet in our study was due to the high intake of plant-derived proteins through the diet.

This is the first study that has evaluated the relation between a maternal MedDiet and total antioxidant content of human breast milk and infant urine. The potential strength of this study is that it evaluates a comprehensive panel of oxidant and antioxidant markers in BM and infant urinary, as well as a wide spectrum of potential confounders variables. Sample collection and data processing were performed with optimal quality control. While the current study has some limitations; since the design of the present study was cross-sectional, definitive causality cannot be expected. To better understand the role of the MedDiet in BM composition, more long-term intervention studies are necessary.

## Conclusion

We have confirmed that the maternal adherence to the MedDiet resulted in the increased levels of TAC (FRAP and DPPH) and protein and the decreased level of TGs in BM, as well as the increased DPPH and FRAP of infant urine. No correlation was found between the maternal diet and levels of MDA, thiol, and calcium in BM and MDA in infant urine. Our results suggest that adhering to a healthy dietary pattern such as MedDiet during pregnancy and breastfeeding may provide a better quality of BM for neonatal nutrition.

## Data Availability

The authors confirm that the data supporting the findings of this study are available.

## Abbreviations

BM: Breast milk
MedDiet: Mediterranean diet
FFQ: Food frequency questionnaire
FRAP: Ferric reducing/antioxidant power
DPPH: 1, 1-Diphenyl-2-picrylhydrazyl
TBARS: Thiobarbituric acid reactive substances
TG: Triglyceride
SIDS: Sudden infant death syndrome
CVD: Cardiovascular disease
FAs: Fatty acids
ROS: Reactive oxygen species
GPx: Glutathione peroxidase
SOD: Superoxide dismutase
TAC: Total antioxidant capacity
BMI: Body mass index
BP: Blood pressure
DBP: Diastolic blood pressure
SBP: Systolic blood pressure
TPTZ: 2,4,6-Tripyridyl-s-triazine
MDA: Malondialdehyde
TBA: Thiobarbituric acid
DTNB: 5,5’-Dithiol-bis-(2-nitrobenzoic acid)
TNB: 5-Thio-2-nitrobenzoic acid
TRL: TG-rich lipoprotein
MUFA/SFA: Monounsaturated to saturated fatty acids
UFAs: Unsaturated fatty acids
SPSS: Statistical package for social sciences
